# Supplementary material for: Impact of body mass index on mortality outcomes in intensive care patients with Staphylococcus aureus sepsis: A retrospective analysis
Source: PLoS One. 2024 Aug 6;19(8):e0308471. doi: 10.1371/journal.pone.0308471 (PMC11302852; doi:10.1371/journal.pone.0308471)
Supplement: Attachment — Submitted filename: Response to Reviewers.docx [file pone.0308471.s001.docx]

Dear Editor and Reviewers:

On behalf of the co-authors, we are very grateful to you for giving us an opportunity to revise our manuscript. We really appreciate your positive and constructive comments together with suggestions on our manuscript entitled ‘Impact of body mass index on mortality outcomes in intensive care patients with S*taphylococcus aureus* sepsis: a retrospective analysis (ID: PONE-D-24-16656).’ We have therefore studied reviewers’ comments carefully and tried our best to revise our manuscript accordingly. Notably, the changes are highlighted in red in the revised manuscript. Please see below for a point-by-point response to the reviewers’ comments and concerns.

**Response to Editor:
Journal Requirements:**

1. Please ensure that your manuscript meets PLOS ONE's style requirements, including those for file naming.

**Response:** The manuscript has been formatted to comply with PLOS ONE style requirements. The documents have been renamed and formatted as per the provided guidelines.

2. Thank you for stating the following financial disclosure: "This work was supported by Hainan Provincial Natural Science Foundation of China.  Project 823RC560." Please state what role the funders took in the study.

**Response:** Funding Statement revised: "This research was supported by the Natural Science Foundation of Hainan Province. Project 823 rc560. The funders had no role in study design, data collection and analysis, decision to publish, or preparation of the manuscript.” The project will also pay a page fee if the article is published**. Please see red fonts on Page 12，line 368.**

3. Thank you for stating the following in your Competing Interests section:  "The authors declare that they have no competing interests."

**Response:** The competing interests section has been updated to state: 'The authors have declared that no competing interests exist.'" **Please see red fonts on Page 12，line 382.**

4. Your ethics statement should only appear in the Methods section of your manuscript. If your ethics statement is written in any section besides the Methods, please move it to the Methods section and delete it from any other section.

**Response:** The ethics statement has been moved to the Methods section and removed from any other sections. **Please see red fonts on Page 3，line 90.**

5. Please ensure that you refer to Figure 2 in your text as, if accepted, production will need this reference to link the reader to the figure.

**Response:** We have ensured that Figure 2 is cited appropriately in the text. **Please see red fonts on Page 8，line 234.**

**Additional Editor Comments:**

Thank you for your submission regarding the relationship between body mass index and mortality in patients with Staphylococcus aureus sepsis using the MIMIC-IV database. However, further clarification in the discussion is need it regarding your findings. For instance, could you try an interpretation regarding type of ventilation in obese and mortality? IS severity score like SOFA II playing a role in prognosis?

**Response:**

Dear Editor,
 Thank you for your valuable feedback on our manuscript regarding the relationship between body mass index (BMI) and mortality in patients with *Staphylococcus aureus* sepsis using the MIMIC-IV database. We appreciate the opportunity to further clarify our findings and incorporate your suggestions.
 We have addressed your comments by revising the discussion section to include an interpretation of the impact of ventilation type on mortality in obese patients and the role of severity scores, such as SOFA II, in prognosis. According to the comments of reviewers, we adjusted the model parameters and performed subgroup analysis. Significant interactions between AKI and SOFA with BMI were observed (P<0.05). These insights have been carefully considered and integrated into our discussion to provide a more comprehensive analysis of our results. We are grateful for your suggestions and are committed to enhancing the quality of our research based on your feedback.

**Revised Discussion Section:**
 In this study, we focused on the short-term mortality of patients with *Staphylococcus aureus* sepsis, specifically within 28 days. Our analysis showed that underweight patients had a significantly increased risk of death. This may be because underweight patients are more likely to experience metabolic imbalances and malnutrition, which can lead to a weakened immune system and overall poorer health, making it more difficult for them to effectively combat severe infections. Similar findings were observed in the study by Lin et al. (2023).
 Previous studies have indicated that MRSA is a significant risk factor for short-term mortality in severe sepsis (Smith et al., 2022; Johnson et al., 2021). Therefore, in this study, we included it as a confounding factor for consideration. However, our findings revealed that the proportion of MRSA did not significantly differ between different BMI groups. This suggests that MRSA positivity is not a major confounding factor in the relationship between BMI and 28-day mortality. Further analysis showed that after controlling for other potential confounders, for each unit increase in BMI, the 28-day mortality decreased by 2.8%. This suggests that a higher BMI provides some protective effect for patients. This phenomenon partly explains the "obesity paradox," where a higher BMI is associated with better outcomes in certain severe illnesses. However, this protective effect is not absolute and can be influenced by other clinical factors.
 For instance, AKI and the SOFA score play important roles in modulating the association between BMI and mortality risk. Our research found that patients with higher BMI and without AKI had a significantly lower risk of death compared to those with AKI. This may be because AKI itself is a serious complication often associated with poorer prognosis, and heavier patients might better mitigate the metabolic burdens associated with AKI.
 Furthermore, the SOFA score significantly affects the association between BMI and mortality. For patients with a SOFA score below 4, a higher BMI is associated with a lower risk of death. In contrast, in cases of multiple organ failure (SOFA score ≥ 4), this protective effect is weakened or even disappears. This indicates that when organ function is not severely compromised, a higher BMI may offer protective benefits; however, in situations of multiple organ failure, this protective effect might no longer exist.
 Regarding the type of ventilation, our analysis suggests that invasive mechanical ventilation is more commonly required in non-overweight patients. However, the increased use of invasive ventilation did not significantly alter the overall mortality risk when adjusting for BMI. This suggests that while ventilation type is an important clinical consideration, it does not independently drive the observed relationship between BMI and mortality.
 We have revised our manuscript accordingly and are confident that these additions will provide a clearer and more thorough understanding of our findings. If you have any further suggestions or questions, please do not hesitate to let us know.

Sincerely,

He-ping Xu
Department of Emergency Medicine, Hainan Affiliated Hospital of Hainan Medical University /Hainan General Hospital, Haikou, Hainan, China.

**Responds to the comments of** **Reviewer #1:**

**Abstract**

**Comment 1:** The statement in the conclusion that "non-invasive ventilation patients with a higher BMI had a lower risk of death compared to invasive ventilation patients" is not supported by the results presented in the manuscript. It is essential to ensure that the conclusions drawn are directly interpretable from the results section. Please revise or substantiate this claim with appropriate data from the results.

**Response:** Thank you for your insightful suggestion. We have thoroughly reviewed the conclusion and acknowledge that the statement regarding "non-invasive ventilation patients with a higher BMI having a lower risk of death compared to invasive ventilation patients" was not explicitly supported by the data presented in the results section. Accordingly, we have removed this part of the conclusion to ensure that our conclusions are directly interpretable from the results presented. **Please see red fonts on Page 2，line 48.**

**revised conclusion:**

Increased BMI is associated with a reduced risk of 28-day all-cause mortality in patients with *Staphylococcus* *aureus* sepsis.

**Introduction**

**Comment 2:** The introduction is currently presented as a single block of text. To improve readability and ensure that the content is easily digestible, please reformat this section into several distinct paragraphs.

**Response:** Thank you for your valuable feedback. I have taken your advice and divided the introduction into three distinct paragraphs to enhance readability and ensure the content is more easily digestible. **Please see the introduction section on page 2.**

**Revised introduction:**

In recent years, the global incidence and prevalence of sepsis have increased significantly (1, 2). Concurrently, obesity remains a prevalent issue worldwide, particularly in the United States where over 25% of adults admitted to intensive care units (ICUs) are classified as overweight, obese, or morbidly obese based on their body mass index (BMI) (3-6). Sepsis is a common reason for these admissions (7-9).

Despite the high prevalence of both conditions, studies exploring the relationship between BMI and the prognosis of sepsis patients are limited. This gap in research highlights the need to investigate the link between BMI and 28-day mortality in patients with sepsis. While obesity is typically associated with increased cardiovascular risks, it may paradoxically offer protective benefits against certain conditions, a phenomenon referred to as the 'obesity paradox.' Observational studies suggest that a higher BMI upon admission is correlated with improved survival and functional outcomes in sepsis patients (10). *Staphylococcus aureus* sepsis, representing the most severe form of *Staphylococcus aureus* infection, accounts for nearly 20% of all hospital-acquired sepsis cases (11, 12). However, the evidence supporting the obesity paradox in cases of *Staphylococcus aureus* sepsis is still emerging.

Therefore, this study aims to explore the relationship between BMI and 28-day mortality in patients suffering from *Staphylococcus aureus* sepsis, which is critical for enhancing clinical decision-making and understanding patient prognosis.

**Methods**

**Comment 3:** You have defined AKI based on serum creatinine levels. However, the definition is incomplete without considering changes in urine output. Please include this information to provide a comprehensive definition of AKI.

**Response:** Thank you for your valuable suggestion. We have revised and improved the diagnostic criteria for Acute Kidney Injury (AKI). AKI is defined as: an increase in serum creatinine of ≥ 0.3 mg/dL (≥ 26.5 μmol/L) within 48 hours, or an increase in serum creatinine to ≥ 1.5 times the baseline level within the past week, or a reduction in urine output to < 0.5 mL/kg/h for 6 hours. **Please see red fonts on Page 4，line 101.**

**Comment 4:** While you mention using the Sepsis-3 criteria for diagnosing sepsis, there is no clarification on how organ dysfunction was confirmed. Please describe the method used to extract and confirm organ dysfunction.

.

**Response:** Your suggestion is very helpful to us. Thank you for your comment. We have revised the diagnostic criteria accordingly. According to the sepsis criteria, organ dysfunction was assessed using the Sequential Organ Failure Assessment (SOFA) score. A SOFA score of 2 or greater was considered indicative of organ dysfunction. The SOFA score evaluates six organ systems (respiratory, cardiovascular, hepatic, coagulation, renal, and neurological) based on clinical parameters and lab results from patient records. **Please see red fonts on Page 4，line 106.**

**Revised content**

According to sepsis-3 criteria, organ dysfunction was assessed using the SOFA score, with ≥2 indicating dysfunction.

**Comment 5:** The choice of variables included in Model 3 of the multivariate regression analysis needs clarification. While including variables related to the patient's condition within 24 hours of ICU admission is understandable, the rationale behind adjusting for ICU and hospital length of stay is unclear. These variables could introduce bias related to the outcome rather than serve as confounders. Please reconsider their inclusion or provide a robust justification.

.

**Response:** Thank you for your insightful comment regarding the variables included in Model 3 of the multivariate regression analysis. We appreciate your concern and have carefully considered your feedback. In response to your suggestion, we have re-evaluated the inclusion of ICU and hospital length of stay in Model 3. Upon further analysis, we have excluded these variables from the model as they may introduce bias related to the outcome, as you rightly pointed out. The revised results, excluding these parameters, are presented in the updated **Table 2 and Figure 4**, reflecting the changes made. For detailed information, please refer to the **revised sections on pages 5, 7, and 8 highlighted in red**. We believe that this adjustment strengthens the robustness of our analysis and addresses the potential bias concerns raised. Thank you for prompting this critical reassessment of our methodology. **Please see red fonts on Page 5,7 and 8, as well as Table 2 and Figure 4.**

**Revised content**

We developed three models: an unadjusted model 1, a model 2 adjusted for age and sex, and a model 3 adjusted for all variables. Using stepwise forward regression, we included variables with a significance threshold of P < 0.05 and excluded those with a significance threshold of P > 0.1. The final model 3 included the following variables: age, diabetes without control, cerebrovascular disease, the first day SOFA score, white blood cell (WBC), hemoglobin, platelets, red cell distribution width (RDW), creatinine, glucose, blood urea nitrogen (BUN), lactate, invasive ventilation, septic shock.

**Results of logistic regression**

Three distinct logistic regression models were developed to evaluate the independent effect of BMI on 28-day all-cause mortality among ICU patients with *Staphylococcus aureus* sepsis (Table 2). Logistic regression analysis indicated an inverse association between BMI and the risk of 28-day all-cause mortality, with an unadjusted odds ratio (OR) of 0.968 (95% CI 0.953–0.983). Using BMI1 as the reference, the crude ORs for BMI2 and BMI3 groups were 0.723 (95% CI 0.551–0.948) and 0.531 (95% CI 0.407–0.692), respectively. After adjusting for age and sex, this inverse association persisted (OR 0.975; 95% CI 0.960–0.991), with adjusted ORs for BMI2 and BMI3 at 0.699 (95% CI 0.529–0.923) and 0.589 (95% CI 0.499–0.773), respectively. Model 3, which incorporated adjustments for age, diabetes without controlled, cerebrovascular disease, first day SOFA score, WBC count, hemoglobin level, platelet count, RDW, creatinine level, glucose level, BUN, lactate level, invasive ventilation, septic shock, showed BMI maintained a strong inverse correlation with 28-day all-cause mortality (OR 0.972; 95% CI 0.955–0.990). The ORs for BMI2 and BMI3 in this model were 0.650 (95% CI 0.474–0.891) and 0.516 (95% CI 0.378–0.705), respectively. Linear trend tests across the models underscored a consistent association between BMI and 28-day mortality.

**Results of subgroup analyses**

To explore potential clinical heterogeneity, we employed interaction and stratification analyses (Figure 4). We examined the association between BMI and 28-day mortality across various subgroups. No significant interactions or differences were observed in stratified analyses based on age (<75 and ≥75 years), sex, MRSA status, renal disease, congestive heart failure, diabetes without control, chronic pulmonary disease, invasive ventilation septic shock, SAPS II score (<37 and ≥37), CCI (<7 and ≥7). Notably, significant interactions between AKI and SOFA with BMI were observed (P<0.05). Non-AKI patients with a higher BMI had a lower risk of death compared to those with AKI. Additionally, patients with a higher BMI and a SOFA score of less than 4 had a lower risk of death than those with a SOFA score of 4 or greater.

Table 2 Relationship between BMI and 28-day all-cause mortality in different models.

|  | Model1  OR (95% CI) P value | | Model2  OR (95% CI) P value | | Model3  OR (95% CI) P value | |
| --- | --- | --- | --- | --- | --- | --- |
| BMI | 0.968 (0.953,0.983) | <0.0001 | 0.975(0.960,0.991) | 0.002 | 0.972(0.955,0.990) | 0.002 |
| BMI |  | |  | |  | |
| BMI1 | Ref | | Ref | | Ref | |
| BMI2 | 0.723(0.551,0.948) | 0.019 | 0.699(0.529,0.923) | 0.012 | 0.650(0.474,0.891) | 0.005 |
| BMI3 | 0.531(0.407,0.692) | <0.0001 | 0.589(0.499,0.773) | <0.0001 | 0.516(0.378,0.705) | <0.0001 |
| P for trend | <0.0001 | | <0.0001 | | <0.0001 | |

OR, odds ratio; CI, confidence interval; Ref, reference; BMI, body mass index MRSA Methicillin-resistant *Staphylococcus aureus*, Model 1 was not adjusted; Model 2 was adjusted for age, sex; and Model 3 was adjusted for age, Diabetes without control, Cerebrovascular disease, First day of SOFA, WBC, Hemoglobin, Platelet, RDW, Creatinine, Glucose, BUN, Lactate, Invasive ventilation, Septic shock.

Figure 4. Effect size of BMI on 28-day mortality in prespecified and exploratory subgroups. The effect size was adjusted for age, Diabetes without control, Cerebrovascular disease, First day of SOFA, WBC, Hemoglobin, Platelet, RDW, Creatinine, Glucose, BUN, Lactate, Invasive ventilation, Septic shock.

**Comment 6:**  The inclusion of AKI should be consistent with the 24-hour post-ICU admission timeline. Additionally, the role of sex as a variable in Model 3 needs clarification regarding its relevance and impact.

**Response:** Thank you for pointing this out. We acknowledge that the inclusion criteria for AKI were not consistent with the 24-hour post-ICU admission timeline. Based on the definition of acute kidney injury (AKI), which includes an increase in serum creatinine by ≥0.3 mg/dL (≥26.5 μmol/L) within 48 hours, we have re-analyzed the AKI cases using the 48-hour data to ensure accuracy and consistency. **Please see red fonts on Page 4，line 101.**

Regarding the inclusion of sex as a variable in Model 3, all potential confounding variables, including sex, were initially included because there was a statistically significant difference in sex among the three BMI groups. We aimed to determine whether sex was a confounding factor between BMI and 28-day mortality. However, through stepwise forward regression, the sex variable was excluded from the final model, indicating that sex had a minimal or non-significant impact in our study. **Please see red fonts on Page 5，line 153.**

**Comment 7:** Please explain the rationale behind setting the age threshold at 75 for subgroup analyses.

**Response:** Thank you again for your valuable feedback. According to the World Health Organization's classification, individuals aged 75 and older are considered elderly. In our study, we set the age threshold at 75 years to differentiate between elderly and non-elderly populations in the subgroup analysis. This age threshold helps us to specifically examine and understand the outcomes in the elderly population. Additionally, several relevant studies in the literature have also used 75 years as the threshold for defining the elderly (e.g., **reference 1)**.

1. Lan W, Liu E, Sun D, Li W, Zhu J, Zhou J, Jin M, Jiang W. Red cell distribution in critically ill patients with chronic obstructive pulmonary disease. Pulmonology. 2024 Jan-Feb;30(1):34-42. doi: 10.1016/j.pulmoe.2022.04.001.

**Results**

**Comment 8:** The use of two different categorizations for BMI (numerical BMI and obesity class) is confusing and could potentially lead to misinterpretation of the data. If obesity class is to be used, please clarify its relevance to the study aims and consider moving detailed data, like those in Table 2, to supplementary content.

**Response:** Thank you for your insightful suggestion. We recognize that using both numerical BMI and obesity categories may cause confusion. To address this, we have standardized our analysis using only the numerical BMI classification. Therefore, we have removed the original Table 2 and related content related to the obesity category to simplify the representation and avoid potential misunderstandings. **Please see red fonts on Page 4，line 98 and Page 7，line 198.**

**Revised content**

BMI (15) is calculated by dividing a person's weight in kilograms by their height in meters squared. Three categories were defined: BMI1 (BMI <25 kg/m²); BMI2 (BMI 25-30 kg/m²); and BMI3 (BMI ≥30 kg/m²).

**Comment 9:** The Kaplan-Meier curve is not mentioned in the Methods section. Please ensure that all statistical methods used in the study are thoroughly described in the Methods section.

**Response:** Thank you for pointing that out. We have now included a detailed description of the Kaplan-Meier survival analysis in the methods section. In order to better clarify the relationship between BMI and 28-day mortality, we used restricted cubic splines to determine thresholds and visualized the nonlinear association between BMI at ICU admission and 28-day mortality. We have added this information to the statistical methods section, **Please see red fonts on Page 6，line 170 and Page 8，line 233.**

**Revised content**

Restricted cubic splines (RCS) were used to determine critical values and visualize the nonlinear association between BMI at ICU admission and 28-day mortality. Kaplan-Meier survival analyses were used to compare the 28-day survival rates of ICU patients with varying BMI levels.

**Results of RCS and** **Kaplan-Meier analysis**

Using restricted cubic splines to determine thresholds, we visualized the nonlinear association between BMI at ICU admission and 28-day mortality, as shown in Figure 2. The analysis revealed a significant nonlinear association between BMI at ICU admission and 28-day mortality in sepsis patients (P = 0.014). When BMI exceeded 28.24 kg/m², the odds ratio for mortality rapidly decreased, but with further increases in BMI, the odds ratio leveled off. Overall, the graph exhibits an "L" shape, indicating that higher BMI levels at ICU admission are associated with lower 28-day mortality odds ratios. Furthermore, Kaplan-Meier curves for different BMI subgroups are presented in Figure 3. The results indicate that higher BMI is associated with lower 28-day mortality (log-rank test, P < 0.0001).

**Comment 10:** The mention of WHO obesity classes is more appropriate for the Results section where classification criteria directly impact the data presentation. Please relocate this information accordingly.

**Response:** Thank you for your valuable feedback. Based on the classification of obesity by the World Health Organization into underweight, normal weight, overweight, and obesity categories, with BMI < 18.5 considered underweight, we have repositioned this information in our study. However, in our study sample, there were only 89 cases with BMI < 18.5, which is a small sample size. Therefore, we have categorized the sample into three groups based on BMI values and decided to remove data related to obesity categories from Table 2 to avoid confusion, focusing solely on categorizing the data by numerical BMI values. **Please see red fonts on Page 4，line 98.**

**Revised content**

Three categories were defined: BMI1 (BMI <25 kg/m²); BMI2 (BMI 25-30 kg/m²); and BMI3 (BMI ≥30 kg/m²).

**Comment 11:** The first character of “staphylococcus aureus” in the title should be capitalized. “Staphylococcus aureus” is usually written in italic as “*Staphylococcus aureus.*”

**Response:** Thank you for your suggestion. The necessary modifications have been made. The first character of "Staphylococcus aureus" in the title has been capitalized, and all instances of “*Staphylococcus aureus*” in the article have been italicized.

**Responds to the comments of Reviewer #2:**

**Comment 1:** A database, MIMIC-IV, is used to extract data. However, the description of this database is scarce, for example, number and kind of ICUs? The sampling encompasses 12 years (2008-2019), what about temporal changes and impact of year of data?

**Response:** Thank you for your insightful comments. We have added a detailed description of the MIMIC-IV database to the manuscript. Please see the updated information on **page 3, line 82.**

The MIMIC-IV (Medical Information Mart for Intensive Care IV) database is a large, freely accessible critical care database, which includes de-identified health-related data from patients admitted to the Beth Israel Deaconess Medical Center in Boston, Massachusetts. It encompasses various types of Intensive Care Units (ICUs), such as medical, surgical, cardiac, and neonatal ICUs.

Regarding the temporal aspect, we regret that we cannot provide data for specific years. The MIMIC-IV database de-identifies patient data to protect privacy, which prevents us from accurately identifying the year of each data entry. This de-identification complies with HIPAA regulations and ensures patient confidentiality.

**Comment 2:** Many ICD-9 and ICD-10 codes is used to incorporate patients with staphylococcus aureus. However, a validation of the codes used is not possible due to the restricted gain to the data: “All data was accessed with read-only permissions to preserve integrity.” Are only patients with a main diagnosis of Staphylococcus aureus sepsis included?

**Response:** Thank you for your insightful comment. In our study, we included any patients diagnosed with sepsis combined with Staphylococcus aureus. We used a comprehensive list of ICD-9 and ICD-10 codes to identify these patients from the MIMIC-IV database.

We apologize for the possible misunderstanding caused by our previous statement: “All data was accessed with read-only permissions to preserve integrity.” In fact, we completed the Citi Program online training course (record ID 59568270), which allows us to extract and download the necessary data for analysis. Therefore, we have revised our statement to: “The required data can be downloaded and analyzed.”

**Please see red fonts on Page 5，line 135.**

**Comment 3:** The BMI groups were alike for several variables, for as example MRSA. But does MRSA depict? MRSA bacteraemia? A local culture positive for MRSA? Please elaborate.

**Response:** Thank you for your insightful comment. In our study, the term MRSA (Methicillin-resistant Staphylococcus aureus) refers to any positive culture for MRSA, whether it is from blood, wound, sputum, or any other body site. We have added this clarification to the manuscript. **Please see the red fonts on Page 4, line 109.** We did not limit our definition to MRSA bacteraemia alone. To clarify, we included patients with any positive culture for MRSA to capture the broad spectrum of MRSA infections, regardless of the specific site of infection. This approach was chosen to ensure a comprehensive analysis of MRSA's impact across different BMI groups.

**Revised content**

Methicillin-resistant Staphylococcus aureus (MRSA) infection refers to any positive culture for MRSA, whether it is from blood, wound, sputum, or any other body site.

**Comment 4:** In the Results section it is said that the highest BMI group developed several conditions. But it is unclear if this Adress condition before the admission or during the admission.

**Response:** Thank you for your insightful comment. In the Results section, the conditions mentioned for the highest BMI group refer to those that were present before hospital admission. We have made the corresponding revisions in the manuscript to clarify this point. **Please see Page 6, line 185 for the updated text.**

**Revised content**

Compared to patients with the lowest BMI, those with the highest BMI are more likely to have comorbidities such as congestive heart failure, chronic lung disease, diabetes without controlled, renal disease, and acute kidney injury. They are also more likely to require continuous renal replacement therapy (CRRT). As BMI increases, both in-hospital mortality and 28-day mortality decrease, as do the average age and proportion of male patients.

**Comment 5:** The Discussion section repeats a number of results but lacks in interpreting these. For example; is the association between BMI and outcome in staphylococcus aureus sepsis due to the pathogen, the organ infection usually caused by the bacterium, or is the association regardless of the pathogen, is the same as for sepsis caused by other pathogens? of course the findings are only associations, but the authors should try to interpret. The Discussion section needs a reframing.

**Response:** Thank you for your insightful feedback. We greatly appreciate your suggestions in the discussion section to strengthen the interpretation of the results. We have revised this section to more thoroughly explore and explain the associations we observed, with a particular focus on the interaction between BMI and *Staphylococcus aureus* sepsis. **Please see Page 9-10, line 283 -313.**

**Here is a revised portion of the discussion:**

In this study, we focused on the short-term mortality of patients with *Staphylococcus aureus* sepsis, specifically within 28 days. Our analysis showed that underweight patients had a significantly increased risk of death. This may be because underweight patients are more likely to experience metabolic imbalances and malnutrition, which can lead to a weakened immune system and overall poorer health, making it more difficult for them to effectively combat severe infections. Similar findings were observed in the study by Lin et al (23).

Previous studies have indicated that MRSA is a significant risk factor for short-term mortality in severe sepsis (24,25). Therefore, in this study, we included it as a confounding factor for consideration. However, our findings revealed that the proportion of MRSA did not significantly differ between different BMI groups. This suggests that MRSA positivity is not a major confounding factor in the relationship between BMI and 28-day mortality. Further analysis showed that after controlling for other potential confounders, for each unit increase in BMI, the 28-day mortality decreased by 2.8%. This suggests that a higher BMI provides some protective effect for patients. This phenomenon partly explains the "obesity paradox," where a higher BMI is associated with better outcomes in certain severe illnesses. However, this protective effect is not absolute and can be influenced by other clinical factors.

For instance, AKI and the SOFA score play important roles in modulating the association between BMI and mortality risk. Our research found that patients with higher BMI and without AKI had a significantly lower risk of death compared to those with AKI. This may be because AKI itself is a serious complication often associated with poorer prognosis, and heavier patients might better mitigate the metabolic burdens associated with AKI.

Furthermore, the SOFA score significantly affects the association between BMI and mortality. For patients with a SOFA score below 4, a higher BMI is associated with a lower risk of death. In contrast, in cases of multiple organ failure (SOFA score ≥ 4), this protective effect is weakened or even disappears. This indicates that when organ function is not severely compromised, a higher BMI may offer protective benefits; however, in situations of multiple organ failure, this protective effect might no longer exist.

**Comment 6:** The references are in general old. The authors should try to incorporate newer ones. Only one example to consider: Alsio¨ Å, Nasic S, Ljungstro¨m L, Jacobsson G (2021) Impact of obesity on outcome of severe bacterial infections. PLoS ONE 16(5): e0251887. https://doi.org/10.1371/journal.pone.0251887

**Response:** Thank you for your feedback regarding the references. We acknowledge that some of the references in our manuscript are dated, and we appreciate your suggestion for incorporating more recent studies. We have reviewed the literature and added newer references to provide a more up-to-date perspective on the topic. Specifically, we have included the study by Alsio et al. (2021), which examines the impact of obesity on the outcome of severe bacterial infections. This addition, along with other recent studies, enhances the relevance and comprehensiveness of our discussion. **Please see Page 12-15, References section.**

Here is the partial reference that has been included:

Alsio¨ Å, Nasic S, Ljungstro¨m L, Jacobsson G (2021). Impact of obesity on outcome of severe bacterial infections. PLOS ONE 16(5): e0251887. https://doi.org/10.1371/journal.pone.0251887

Sjöwall C. Obesity and tobacco smoking are independently associated with poor patient-reported outcomes in SLE: a cross-sectional study. Rheumatol Int. 2024;44(5):851-61. http://doi.org/10.1007/s00296-024-05546-z

Lebovitz S, Rozen G, Abu GZ, Korem M, Elinav H, Zayyad H, et al. The Relationship between Body Mass Index and In-Hospital Mortality in Bacteremic Sepsis. J Clin Med. 2023;12(11). http://doi.org/10.3390/jcm12113848

MRSA Infections In Chongqing, China. Infect Drug Resist. 2019; 12:3709-17. http://doi.org/10.2147/IDR.S223536

Wong JW, Ip M, Tang A, Wei VW, Wong SY, Riley S, et al. Prevalence and risk factors of community-associated methicillin-resistant Staphylococcus aureus carriage in Asia-Pacific region from 2000 to 2016: a systematic review and meta-analysis. Clin Epidemiol. 2018; 10:1489-501. http://doi.org/10.2147/CLEP.S160595

**Comment 7:** Some patients do not have data for height and length. How many and is there a need for a dropout-analysis?

**Response:** I apologize for the confusion caused by the initial comment. We have revised the original text to clarify the exclusion criteria. The third criterion now reads, "those lacking height or weight data, essential for accurate BMI calculation." Patients without height or weight data are excluded from the analysis. **Please see Page 4, line124.**

**Comment 8:** The association between BMI and outcome in staphylococcus aures sepsis is sometimes described as a linkage in the manuscript. Is this a proper term?

**Response:** Thank you for pointing that out. I have made revisions to the entire manuscript. For example, in the abstract section, the wording is now as follows: "Evidence associating body mass index (BMI) with the prognosis of *Staphylococcus aureus sepsis* remains scarce." This revision better reflects the relationship between BMI and the prognosis of *Staphylococcus aureus* sepsis. **Please see Page 1, line17.**

**Comment 9:** The age limit is 18 y or 19 y?

**Response:** I apologize for the confusion in the wording of the manuscript. We have made the necessary adjustments to clarify the age limit. The revised statement now reads: "The inclusion criteria for this study encompassed patients aged 18 years and older who were diagnosed with Staphylococcus aureus sepsis during their ICU stay." The age criterion for inclusion is 18 years and older. Thank you for bringing this to our attention. **Please see Page 4, line113.**

**Comment 10:** What is the rationale for including the variable red cell distribution width (RDW)?

**Response:** Thank you for your question regarding the inclusion of red cell distribution width (RDW) as a variable in our study. The rationale for incorporating RDW as a variable in our analysis is based on emerging evidence suggesting that RDW is a measure of variability in red blood cell size and can serve as a prognostic marker for various medical conditions, including sepsis. Elevated RDW levels have been associated with increased mortality and poor outcomes in septic patients [1-5].

Therefore, by including RDW as a variable in our study, we aim to explore its potential role as a prognostic indicator in patients with Staphylococcus aureus sepsis. We believe that investigating the relationship between RDW and sepsis outcomes may provide valuable insights into the pathophysiology and prognosis of this condition. Thank you for your interest in our research.

References:

1. Lorente L, et al. Red blood cell distribution width during the first week is associated with severity and mortality in septic patients. PLoS One. 2014.

2. Wu H, et al. Diagnostic value of RDW for the prediction of mortality in adult sepsis patients: A systematic review and meta-analysis. Front Immunol. 2022.

3.Hu ZD, et al. Diagnostic and prognostic value of red blood cell distribution width in sepsis: A narrative review. Clin Biochem. 2020.

4. Li Y, et al. Association Between Red Cell Distribution Width and Hospital Mortality in Patients with Sepsis. J Int Med Res. 2021.

5. Han YQ, et al. Red blood cell distribution width predicts long-term outcomes in sepsis patients admitted to the intensive care unit. Clin Chim Acta. 2018.

**Comment 11:** Why not including information about lymphocyte count and the neutrophil-lymphocyte count ratio, as it has been proposed to be a factor differentiating the inflammatory response between the obese and normal weights?

**Response:** Thank you for highlighting the potential importance of including information about lymphocyte count and the neutrophil-lymphocyte count ratio in our study. We appreciate your suggestion and acknowledge the significance of these factors in differentiating the inflammatory response between obese and normal-weight individuals.

In the context of sepsis, lymphocyte count and the neutrophil-lymphocyte count ratio are indeed valuable indicators of the immune response and can provide insights into the severity and prognosis of the condition. These parameters have been shown to be associated with outcomes in septic patients and may play a role in predicting mortality and morbidity.

While our study focused specifically on the relationship between BMI and sepsis, information on lymphocyte count and the neutrophil-lymphocyte count ratio may be potential confounding factors that were not included in our analysis. However, we recognize the potential value of incorporating additional inflammatory markers in future research. Including these parameters could provide a more comprehensive evaluation of the inflammatory response in sepsis patients, particularly in the context of obesity.

We appreciate your input and will consider including information on lymphocyte count and the neutrophil-lymphocyte count ratio in future studies to further elucidate the relationship between inflammatory markers and outcomes in sepsis patients, especially in different weight categories. Thank you for your valuable suggestion.
